# Case Report: Treatment of an unresectable mucoepidermoid carcinoma with osimertinib

**DOI:** 10.3389/fonc.2026.1927746

**Published:** 2026-09-11

**Authors:** Leonardo Villa, Alice Soares, Viviane Basílio, Haonne Abboud, Daniel Herchenhorn

**Affiliations:** Department of Medical Oncology, D’Or Institute for Research and Education (IDOR), Rio de Janeiro, Brazil

**Keywords:** case report, EGFR, mucoepidermoid carcinoma, osimertinib, salivary gland cancer, tumor-agnostic therapy

## Abstract

**Background:**

Mucoepidermoid carcinoma (MEC) is a common salivary gland malignancy, yet no established systemic standard exists for unresectable disease, and response rates to conventional chemotherapy are low. Most MECs are driven by the CRTC1–MAML2 fusion, which induces amphiregulin (AREG) overexpression and an autocrine AREG–EGFR loop that hyperactivates EGFR independently of activating kinase-domain mutations, providing a rationale for tumor-agnostic EGFR blockade.

**Case presentation:**

A 72-year-old man presented with a slowly enlarging right submandibular mass, diagnosed as low-grade MEC of the floor of the mouth. The tumor was considered unresectable owing to anticipated functional and cosmetic morbidity, and radiotherapy was deferred because of extensive mandibular involvement. Comprehensive genomic profiling identified no high-tier actionable alteration and did not interrogate gene fusions. On the basis of the AREG–EGFR autocrine axis described in MEC, osimertinib 80 mg once daily was initiated. The patient achieved a 33% reduction in the longest tumor diameter, meeting RECIST 1.1 criteria for a partial response, with symptomatic improvement and only grade 1 toxicity. Osimertinib was maintained at 80 mg once daily throughout, with approximately seven and a half months of disease control before non-target progression prompted palliative radiotherapy, for a total of 13 months of continuous treatment on this tumor-agnostic strategy. The patient remained clinically stable at last follow-up.

**Conclusion:**

To our knowledge, this is the first reported partial response to a third-generation EGFR-TKI in oral MEC. The case supports prospective evaluation of fusion-informed, EGFR-directed therapy in this rare malignancy.

## 1 Introduction

Mucoepidermoid carcinoma (MEC) is the most common malignant salivary gland tumor, accounting for approximately 10-15% of all salivary gland neoplasms (1); salivary gland cancers as a whole, however, are rare. Its management currently rests on surgical excision of resectable tumors, with systemic therapy, chemotherapy, and radiotherapy reserved for advanced disease (1). In unresectable cases, however, establishing an evidence-based treatment standard with good external validity is difficult because of the scarcity of patients available for randomized clinical trials; the few studies conducted to date have shown very low response rates. This has motivated interest in identifying genetic targets that enable tumor-agnostic therapy aimed at improving survival in patients affected by this disease (1).

Recently identified chromosomal and proteomic abnormalities—including CDKN2A, TP53, CDKN2B, BAP1, and, in particular, EGFR—are involved in the transformation of low-grade into high-grade tumors and may represent potential therapeutic targets (1). In the pulmonary variant of this disease, EGFR protein overexpression has been common in most cases, without amplification or tyrosine kinase–domain mutation of the originating gene. This feature has positioned EGFR-blocking targeted therapy, such as gefitinib and erlotinib, as an emerging option for patients with metastatic pulmonary MEC despite the absence of a sensitizing mutation (2).

Another highly significant alteration in these tumors is the CRTC1/3-MAML2 fusion, detected via FISH and RT-PCR. This alteration, resulting from the t (11,19)(q21;p13) translocation, is considered the primary oncogenic driver in MEC (3). Its reported prevalence in salivary gland MEC ranges from 58.8% to 62.4% (4, 5), and is even higher in young patients—reaching 82% in those under 30 years of age (4), according to a Japanese multicenter series. This fusion activates downstream pathways, including AREG-EGFR signaling, rendering fusion-positive MEC cells highly sensitive to EGFR inhibition (6).

We report a case, drawn from the medical-record review of a private clinic, in which this tumor-agnostic rationale motivated the use of a third-generation tyrosine kinase inhibitor (osimertinib) in a patient with unresectable oral disease. The patient achieved an objective, sustained partial response maintained to date—to our knowledge the first reported response to a third-generation EGFR-TKI in oral MEC.

## 2 Case description

A 72-year-old man presented with a right submandibular nodule of approximately 5 years’ duration. He had initially undergone fine-needle aspiration, which yielded benign cytologic findings. The patient reported a prior fistula and multiple dental interventions in 2024. The lesion enlarged slowly over time and was subsequently evaluated by an oral and maxillofacial surgeon. In November 2024, an incisional biopsy demonstrated low-grade mucoepidermoid carcinoma (MEC).

The patient was referred to a head and neck surgery service, where surgical resection was initially recommended requiring mandibulectomy and excision of a large portion of buccal mucosa; a complete resection would not be possible due to extensive muscle extension requiring plastic reconstruction and with a high probability of severe chronic morbidities. The patient declined surgery because of the anticipated functional and cosmetic morbidity, and alternative treatment options were then considered. His medical history was notable for systemic arterial hypertension treated with indapamide and losartan. Family history included prostate cancer in his father and breast cancer in his sister. He had no history of tobacco or alcohol use and maintained regular physical activity, including walking and resistance training.

On examination, the patient was in good general condition, with an ECOG performance status of 1 and a Karnofsky Performance Status of 90%. A large, indurated mass involving the floor of the mouth, measuring approximately 5 × 5 cm, was present with evidence of local invasion. Because surgery had been refused, definitive-intent radiotherapy was considered but deferred by the multidisciplinary team, as irradiating a lesion with mandibular cortical erosion and altered marrow signal to the required 66–70 Gy carried a high risk of osteoradionecrosis. The remainder of the physical examination was unremarkable. Body weight was 55 kg, with a body mass index of 19.7 kg/m². The patient reported no pain at presentation.

## 3 Diagnostic assessment

### 3.1 Pathology

The November 2024 incisional biopsy sampled oral mucosa, right submandibular soft tissue, and mandibular bone from the angle and body of the mandible. Histopathologic examination demonstrated low-grade MEC infiltrating both bone and adjacent soft tissues. The tumor exhibited a predominantly cystic architecture composed of mucous, intermediate, and epidermoid cells, with minimal cytologic atypia and low mitotic activity, consistent with a low-grade neoplasm according to established grading systems. Perineural invasion and lymphovascular invasion were not identified, and tumor necrosis was absent. One sampled lymph node was negative for metastatic disease. Representative histopathologic sections are shown in **Figure 1**.

**Figure 1:**
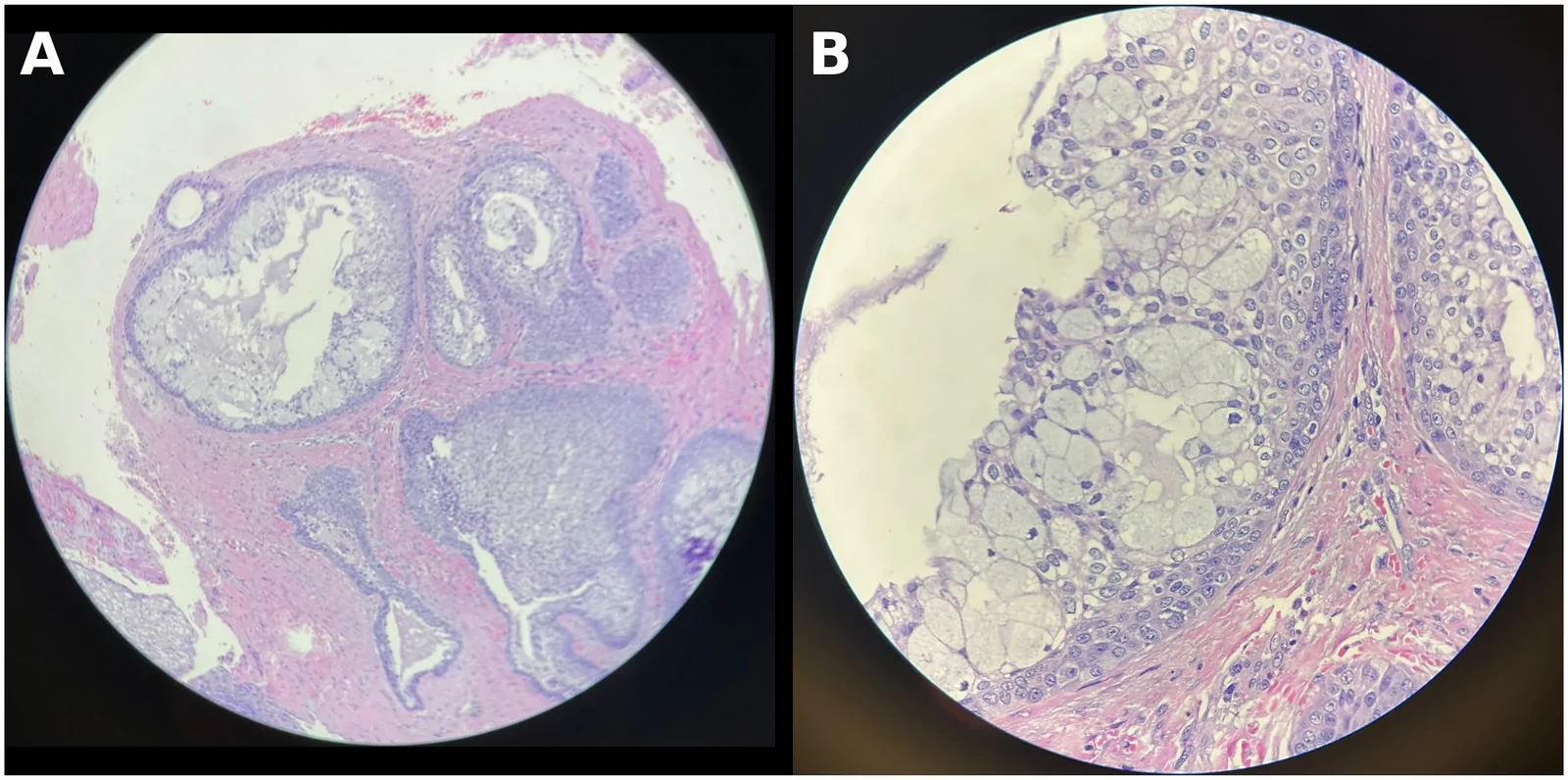
Histopathologic features of the low-grade mucoepidermoid carcinoma on the November 2024 incisional biopsy (hematoxylin and eosin). **(A)** Low-power view showing a predominantly cystic architecture. **(B)** High-power view showing mucous, intermediate and epidermoid cells, with minimal cytologic atypia and low mitotic activity. Perineural and lymphovascular invasion were not identified and tumor necrosis was absent.

### 3.2 Imaging and staging

In January 2025, magnetic resonance imaging (MRI) demonstrated a 6.6 × 5.0 cm lesion centered within the right sublingual space, associated with mandibular cortical erosion and displacement of adjacent musculature. A positron emission tomography/computed tomography (PET/CT) scan performed during the same period demonstrated a 5.7 cm mass with heterogeneous fluorodeoxyglucose uptake (SUVmax 7.4) and no evidence of distant metastatic disease. Tumor measurements were obtained from contrast-enhanced T1-weighted axial MRI sequences. Representative cross-sectional imaging at baseline and at best response is shown in **Figure 2**.

**Figure 2:**
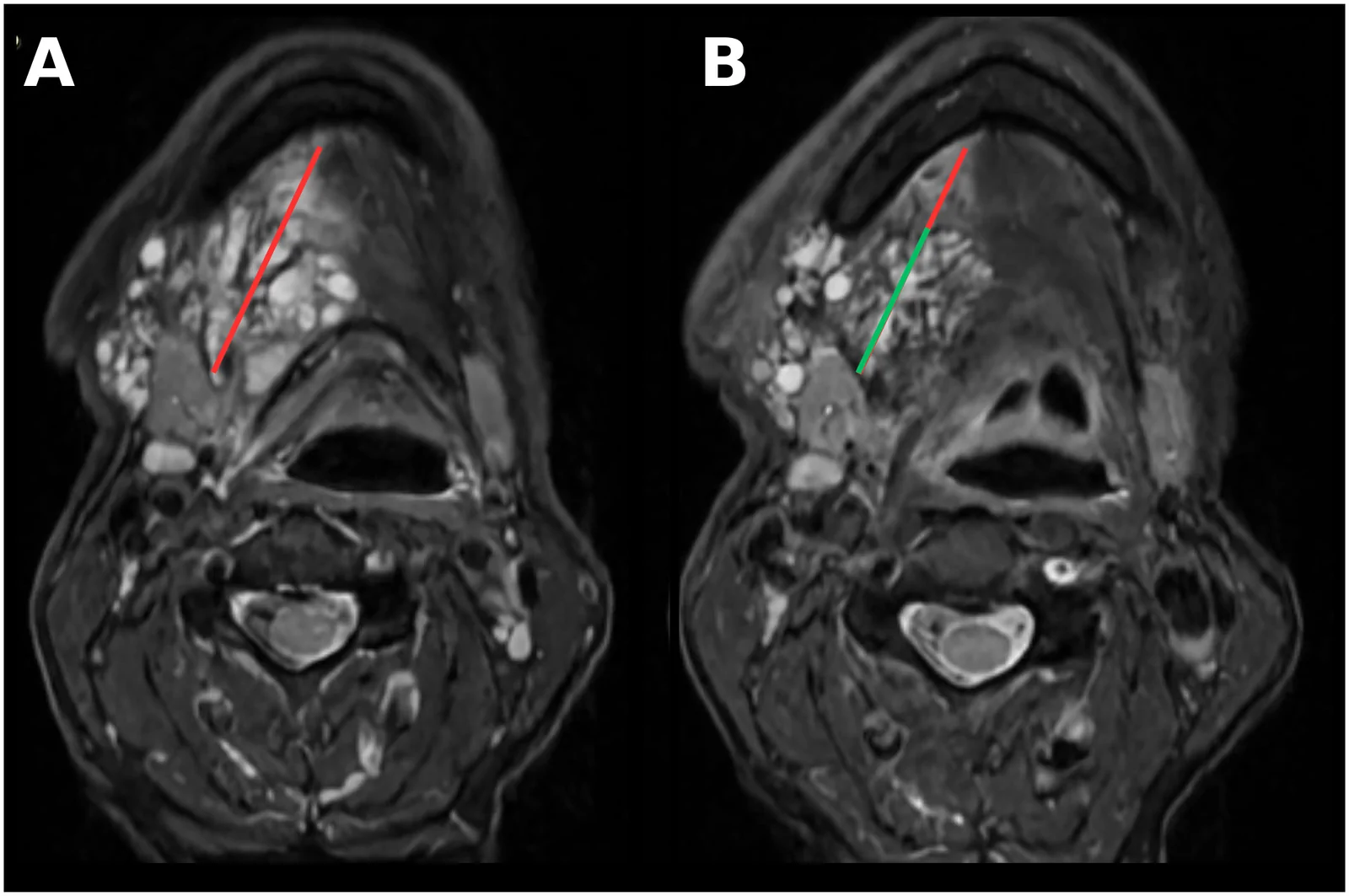
Contrast-enhanced T1-weighted axial MRI of the index lesion. **(A)** Baseline (March 2025), longest diameter 8.2 cm (red line). **(B)** Post-treatment (July 2025), longest diameter 5.5 cm (green line), a 33% reduction meeting RECIST 1.1 criteria for partial response; the baseline diameter is reproduced in red for comparison. The lesion is centered in the right sublingual and submandibular space, with mandibular cortical erosion and posteroinferior displacement of the right submandibular gland, which is secondarily involved rather than the site of origin.

### 3.3 Molecular profiling

Comprehensive genomic profiling using a 500-gene DNA panel identified no high-tier actionable genomic alterations. HER2 (ERBB2) immunohistochemistry was scored as 2+, without evidence of gene amplification by fluorescence *in situ* hybridization (FISH). Estrogen receptor and progesterone receptor expression were negative. A tier 2C KRAS G13D (p.Gly13Asp) variant was detected but was considered of uncertain therapeutic significance.

A key limitation of the molecular analysis was that the assay did not evaluate gene fusions. Consequently, CRTC1–MAML2 fusion status could not be determined. Fusion testing by RNA sequencing, FISH, reverse-transcription polymerase chain reaction (RT-PCR), or RNA *in situ* hybridization was not included in the available panel and was not accessible in our region. The assay performed was a targeted DNA-based panel, which detects structural rearrangements reliably only when the relevant intronic breakpoint regions are specifically tiled; CRTC1–MAML2 is therefore not interrogated by such panels even when the fusion is present, and is identified dependably only by RNA-based or cytogenetic methods. In addition, none of these fusion-directed assays is available at our institution or through the reference laboratories serving it, and this remained the case throughout the disease course: fusion status could not be determined at diagnosis, at the time of the treatment decision, or at repeat profiling in April 2026.

## 4 Therapeutic intervention, follow-up, and outcomes

### 4.1 Treatment rationale

Two considerations governed the decision not to pursue local treatment first. Surgery had been recommended but would have required mandibulectomy with excision of a large portion of the buccal mucosa and plastic reconstruction, with a high probability of severe permanent functional and cosmetic morbidity in a 72-year-old man; complete resection was judged unlikely given the extent of muscular involvement, and the patient declined. Radiotherapy was not refused by the patient but deferred on clinical grounds, as detailed above.

With no established systemic standard for unresectable MEC and low response rates to conventional chemotherapy, systemic therapy was therefore selected as the approach least likely to preclude subsequent local treatment. The partial response subsequently obtained was not judged by the surgical team to be sufficient to reduce the extent of a potential resection.

Based on a tumor-agnostic therapeutic strategy targeting the amphiregulin (AREG)–EGFR autocrine signaling axis described in MEC, treatment with an EGFR tyrosine kinase inhibitor (EGFR-TKI) was pursued despite the absence of an activating EGFR mutation.

Among EGFR-TKIs previously reported in MEC, including gefitinib and erlotinib, osimertinib was selected because of its favorable toxicity profile, lower rates of severe cutaneous and gastrointestinal adverse events, and convenient once-daily oral administration. These characteristics were considered particularly relevant for a 72-year-old patient receiving treatment with non-curative intent while prioritizing quality of life. Osimertinib 80 mg once daily was initiated on 28 March 2025 after a multidisciplinary tumor board discussion with the presence of specialized oncologists and geneticists.

### 4.2 Response assessment and clinical course

Treatment response was assessed according to RECIST version 1.1 using a single target lesion. Because interval tumor growth occurred between January and March 2025, the March 2025 MRI was designated as the RECIST baseline assessment. The January 2025 scan preceded osimertinib initiation by more than four weeks and was therefore not used for response evaluation. Baseline MRI demonstrated a lesion measuring 8.2 × 5.8 × 6.7 cm, with persistent evidence of mandibular invasion.

Follow-up MRI in July 2025 demonstrated substantial tumor regression, with dimensions of 5.5 × 4.5 × 5.0 cm and a longest diameter of 5.5 cm. This represented a 33% reduction in the longest diameter relative to baseline and met RECIST 1.1 criteria for a partial response (**Figure 2**; **Supplementary Table 1**).

Clinically, the patient reported symptomatic improvement, including reduced oropharyngeal secretions and a subjective sensation of tumor regression; the corresponding local response of the ulcerated, fistulizing component is shown in **Figure 3**. Treatment-related adverse events were limited to grade 1 folliculitis of the lower extremities, which was controlled with topical therapy, and mild asthenia. Laboratory testing performed in July 2025 was within normal limits. Serum CA19–9 decreased from 127 U/mL in March 2025 to 73 U/mL in July 2025, although the clinical significance of this finding remains uncertain.

**Figure 3:**
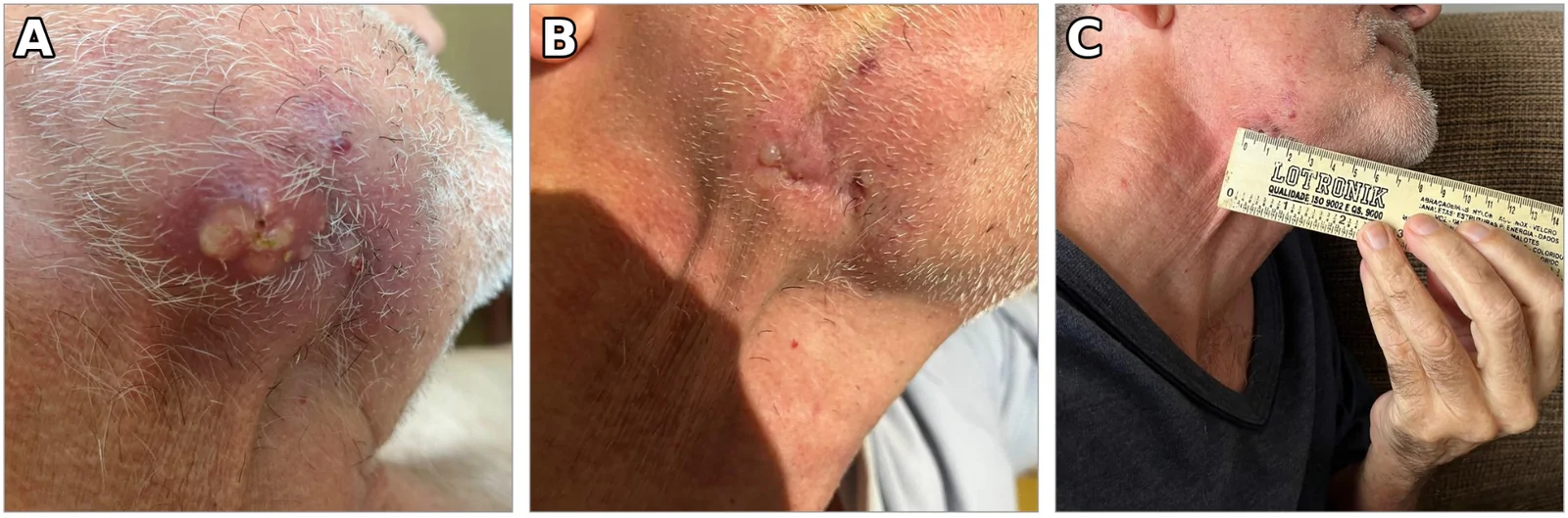
Local response of the unresectable oral mucoepidermoid carcinoma to osimertinib (80 mg, one tablet daily, since March 2025). **(A)** Baseline ulcerated, fistulizing lesion of the right submandibular region before treatment. **(B)** Partial healing of the lesion during osimertinib therapy. **(C)** Local response observed in July 2025, with the residual lesion measured against a ruler.

Disease control was maintained until November 2025. Follow-up MRI on 10 November 2025 demonstrated a slight increase in the longest target-lesion diameter from 5.5 cm to 6.0 cm (+9% relative to the July nadir), which did not by itself meet RECIST 1.1 criteria for target-lesion progression; no new lesions were identified. However, the report described greater cutaneous superficialization of the anteroinferior component of the lesion extending into the submental region, together with areas of restricted diffusion that were less intense than on the previous study and internal cystic/necrotic degeneration—findings compatible with a persisting treatment effect. Despite physical examination in the same month showing reduction of the ulcerated aspect of the lesion, a vegetating and exophytic component had appeared, of a morphology not present at any earlier assessment, associated with a poorly defined submental mass—corresponding on MRI to anteroinferior extension of the index lesion rather than to a discrete new lesion—that was fixed to the underlying musculature and painless.

Cutaneous toxicity was considered unlikely to account for the latter: the only dermatologic adverse event attributable to osimertinib was a grade 1 folliculitis confined to the lower extremities; the dose remained unchanged at 80 mg once daily throughout, so no dose–lesion temporal relationship could be established; and no local therapy had been applied to that skin at any time.

The event was therefore classified as progressive disease by RECIST 1.1. The target lesion remained stable, but the vegetating cutaneous component — non-target disease distinct from the pre-existing ulcerated, fistulizing area, which had improved and remained improved — showed unequivocal progression clearly attributable to tumor. Management options were discussed with the patient; biopsy was not pursued, as the clinical course was considered unequivocal. Osimertinib was maintained at 80 mg once daily and the patient was referred to radiotherapy. Treatment consisted of 55 Gy delivered in 20 fractions with palliative and local-control intent, completed in January 2026 without reported treatment-related toxicity. The treating radiation oncologist documented a subjective response of the vegetating component of the irradiated lesion, although this was not formally quantified by RECIST.

At the most recent follow-up in April 2026, the patient maintained clinical and radiologic stability, with continuous use of osimertinib. Repeat genomic profiling similarly failed to identify actionable genomic alterations; however, CRTC1–MAML2 fusion testing remained unavailable. The patient preserved excellent functional status throughout treatment, with ECOG performance status of 0, remained under nutritional follow-up, and was scheduled for repeated MRI and clinical evaluation every three months. The chronological course of care is summarized in the clinical timeline (**Table 1**).

**Table 1:** Chronological summary of the clinical course (CARE timeline).

Date	Clinical milestone	Imaging/laboratory findings	Treatment and outcome
Nov 2024	Diagnosis	Incisional biopsy: low-grade mucoepidermoid carcinoma (right submandibular/floor of mouth; ~5-year history; prior benign FNA).	Referred to head and neck surgery.
Jan 2025	Staging; unresectable disease	MRI 6.6 × 5.0 cm, right sublingual space, mandibular cortical erosion. PET-CT 5.7 cm, SUVmax 7.4; no distant metastases.	Mutilating surgery declined (high morbidity); radiotherapy deferred (osteonecrosis risk). Incidental Gleason 6 prostate adenocarcinoma → active surveillance.
Mar 2025	Baseline; osimertinib started	MRI 8.2 × 5.8 × 6.7 cm (longest diameter 8.2 cm) — interval growth; designated RECIST 1.1 baseline.	Osimertinib 80 mg/day started 28 Mar 2025 (tumor-agnostic EGFR blockade).
Jul 2025	Partial response (RECIST 1.1; best overall response)	MRI 5.5 × 4.5 × 5.0 cm (longest diameter 5.5 cm): 33% decrease vs baseline → partial response (nadir). CA19-9 127 → 73 U/mL; other labs normal.	Symptomatic improvement; grade 1 folliculitis and mild asthenia; ECOG 0. Osimertinib continued.
Nov 2025	Progressive disease (RECIST 1.1) by unequivocal non-target progression	MRI (10 Nov 2025) 5.5 × 4.5 × 6.0 cm (longest diameter 6.0 cm): +9% vs July nadir — does NOT by itself meet RECIST 1.1 criteria for target-lesion PD; no new lesions. Greater cutaneous superficialization of the anteroinferior component extending into the submental region; restricted diffusion less intense than on the previous study; Examination: submandibular nodule and pre-existing ulcerated area improved; new vegetating, exophytic component (non-target) with unequivocal progression; poorly defined submental mass fixed to the underlying musculature, painless.	Osimertinib maintained at 80 mg/day; referred for radiotherapy.
Jan 2026	Radiotherapy	—	55 Gy in 20 fractions, palliative intent; no reported side effects; marked subjective local response documented.
Apr 2026	Last follow-up	Repeat genomic panel: no actionable mutations (fusion not interrogated).	Clinically stable; ECOG 0. MRI and clinical examination scheduled in 3 months.

FNA, fine-needle aspiration; MRI, magnetic resonance imaging; PET-CT, positron emission tomography–computed tomography; SUVmax, maximum standardized uptake value; EGFR, epidermal growth factor receptor; ECOG, Eastern Cooperative Oncology Group; RECIST, Response Evaluation Criteria in Solid Tumors; CA19-9, carbohydrate antigen 19-9.

## 5 Discussion

The mutation-independent response observed here is mechanistically explained by the central molecular event of MEC: the t (11, 19) translocation generating the CRTC1–MAML2 fusion (and, less frequently, CRTC3–MAML2) (3). In a conditional transgenic model the fusion behaved as the principal oncogenic driver, inducing salivary gland tumors with complete penetrance, while its silencing blocked established xenograft growth (3).

The fusion co-activates CREB and induces amphiregulin (AREG), generating an AREG→EGFR autocrine loop that sustains MEC growth and survival; EGFR is thus hyperactivated by ligand overexpression rather than by a kinase-domain mutation (**Supplementary Figure 1**) (6).

As noted above, the fusion is present in up to 62.4% of MECs and in approximately 82% of patients up to 30 years of age (4, 5), and it also carries diagnostic and prognostic weight. It may help distinguish true MEC from poorly differentiated oral lesions (7), and a meta-analysis links it to better survival (8), although individual cohorts have not confirmed it as an independent prognostic factor, favoring instead grade, nodal status, distant and intraparotid metastasis, and age (9). EGFR tyrosine kinase–domain mutations are rare in MEC and other salivary carcinomas (10); in a series of 101 cases, EGFR-pathway mutations (RAS/PIK3CA) occurred in 14.9% and TP53 in 20.8%, with the fusion associated with better and RAS/PIK3CA with worse prognosis (11). Cell-line studies suggest that EGFR dependence may diminish at recurrence or progression (12), consistent with the limited duration of benefit observed here.

Our experience should be read against the limited literature on EGFR-directed therapy in MEC. Gefitinib has produced anecdotal responses in pulmonary MEC lacking sensitizing EGFR mutations, including a pathologic complete response in a child with EGFR-negative low-grade pulmonary MEC (13), with the CRTC1–MAML2/AREG axis proposed as the mechanism (14); rare drug-sensitizing EGFR exon 19 deletions have also been reported in parotid MEC (15). In the salivary setting, however, prospective data are discouraging: a phase II trial of gefitinib in molecularly unselected advanced salivary gland cancers reported no objective responses, including among MEC patients (16). Antibody-mediated EGFR blockade has fared no better: a phase II study of cetuximab in 30 unselected patients with recurrent or metastatic salivary gland carcinomas produced no objective response, the clinical-benefit rate of 50% being driven entirely by disease stabilization (17). By contrast, molecularly matched approaches have been more encouraging when the target is selected for. In the MyPathway basket study, HER2-directed pertuzumab plus trastuzumab achieved a 60% objective response rate in HER2-amplified/overexpressing salivary cancers (18), and the NCI-MATCH program demonstrated that basket trials can uncover tumor-specific activity even when an agent lacks tissue-agnostic efficacy (19).

Against this background, a 33% RECIST partial response to a third-generation EGFR-TKI in oral MEC—to our knowledge previously unreported—is notable, and the generation of inhibitor chosen bears directly on tolerability. Osimertinib carries a substantially lower burden of severe cutaneous and gastrointestinal toxicity than the first-generation agents previously reported in MEC, a profile that was decisive in a 72-year-old man treated with non-curative intent. This was borne out here: the only dermatologic adverse event was a grade 1 folliculitis of the lower extremities, remote from the tumor bed and stable at an unchanged dose.

From a practical standpoint, systematic reviews reinforce that response to systemic therapy depends heavily on histology and molecular target, and that a personalized approach based on molecular screening is the most promising avenue (20). Incorporating RNA *in situ* hybridization—highly accurate for identifying CRTC1–MAML2 (21)—into the workup of unresectable or metastatic MEC therefore warrants consideration, as it could select candidates for EGFR blockade regardless of actionable somatic or germline mutations on conventional testing.

The sequence of local and systemic treatment in this case also merits comment. Radiotherapy had initially been deferred because of the risk of osteoradionecrosis in a bulky lesion with mandibular cortical erosion. The volume irradiated in January 2026 measured 6.0 cm, compared with 8.2 cm before systemic treatment, and 55 Gy in 20 fractions were delivered with palliative intent, without reported treatment-related toxicity and without osteoradionecrosis, with a marked subjective local response. Whether the cytoreduction obtained with osimertinib rendered subsequent irradiation more feasible cannot be established from a single case, and this observation should be regarded as hypothesis-generating rather than demonstrated; it does, however, argue against the interpretation that systemic therapy merely delayed local treatment.

A final point concerns access. Osimertinib is not approved for salivary gland malignancy by any regulatory agency, and its use here was off-label clinical care rather than research: the decision was taken at a multidisciplinary tumor board, the absence of any standard systemic option was documented, and written informed consent for off-label treatment was obtained. The drug was not supplied by the manufacturer but funded by the patient’s private health insurer, through prior authorization of an off-label use of a nationally registered agent. Access elsewhere should be expected to depend on local regulation and payer policy rather than on any disease-specific program.

### 5.1 Strengths and limitations

The principal strength of this report is the prospectively documented, RECIST-assessed objective response with serial imaging and a clearly defined baseline, in a setting where therapeutic options are scarce. This report also has important limitations. First and foremost, CRTC1-MAML2 fusion status—the molecular cornerstone of the proposed mechanism—could not be determined, as fusion testing (RNA sequencing, FISH, RT-PCR, or *in situ* hybridization) was neither covered by the available genomic panel nor accessible in our region; the EGFR-directed rationale therefore rests on the high prevalence of this fusion in MEC reported in the literature rather than on direct confirmation in this patient. The single-patient design precludes any inference of efficacy, and the benefit was of limited duration, consistent with the diminishing EGFR dependence reported in preclinical models. Finally, progression was declared on unequivocal worsening of a non-target cutaneous component assessed clinically; although the divergent behavior of the two cutaneous components and the radiologic finding of increased cutaneous superficialization support attribution to tumor, non-target assessment is qualitative by definition and carries a degree of observer dependence that a measurable target-lesion threshold does not.

### 5.2 Take-away lessons

In unresectable MEC without an established systemic standard, a tumor-agnostic EGFR-directed strategy grounded in the AREG–EGFR autocrine biology of the disease produced a meaningful, objective, and well-tolerated response. The case argues for incorporating fusion testing (ideally RNA *in situ* hybridization) into the standard diagnostic workup of advanced MEC rather than treating it as an ancillary test, so as to identify candidates for EGFR blockade, and for prospective, fusion-informed trials. In conclusion, this case—the first reported partial response to a third-generation EGFR-TKI in oral MEC—highlights the need for prospective trials evaluating EGFR-directed, fusion-informed therapy and the best candidates for it, to advance treatment of this challenging disease.

## 6 Patient perspective

The patient expressed satisfaction at being included in this case report, noting that sharing his experience could contribute to the discussion of new therapeutic possibilities for his underlying malignancy and might help other patients facing the same rare diagnosis.

## Data Availability

All data supporting the conclusions of this article are included in the article and its **Supplementary Material**. Further inquiries can be directed to the corresponding author. Individual patient data are not publicly available in order to protect patient privacy.

## Supplementary material

Supplementary Figure 1 Schematic of CRTC1–MAML2 fusion–driven AREG–EGFR signaling in mucoepidermoid carcinoma. The fusion coactivates CREB and induces amphiregulin (AREG), generating an autocrine loop that hyperactivates EGFR through ligand overexpression rather than through a tyrosine kinase–domain mutation. Downstream, the JAK-STAT and MAPK/ERK pathways are activated; in parallel, the p16–CDK4/6–RB–E2F axis cooperates in cell-cycle entry. EGFR tyrosine kinase inhibitors (gefitinib, erlotinib, osimertinib) and the CDK4/6 inhibitor (palbociclib) indicate the points of therapeutic blockade.

Supplementary Table 1 RECIST 1.1 target-lesion measurements over the treatment course. Longest diameter of the single target lesion at baseline (March 2025), at best response (July 2025) and at the November 2025 assessment, with the corresponding percentage change from baseline and from nadir.
