# Supplementary material for: Case Report: Treatment of an unresectable mucoepidermoid carcinoma with osimertinib
Source: Front Oncol. 2026 Sep 11;16:1927746. doi: 10.3389/fonc.2026.1927746 (PMC13612365; doi:10.3389/fonc.2026.1927746)
Supplement: Supplementary file 1 [file Table1.docx]

Supplementary Material

**Supplementary Table S1.** RECIST 1.1 target-lesion measurements and response category over time.

| **Timepoint** | **Modality / sequence** | **Target lesion longest diameter (cm)** | **% vs baseline** | **% vs nadir** | **RECIST 1.1 category** |
| --- | --- | --- | --- | --- | --- |
| Jan 2025 | MRI, post-contrast T1 | 6.6 | — | — | Pre-baseline (not used; >4 wk before therapy) |
| Mar 2025 (baseline) | MRI, post-contrast T1 | 8.2 | 0% | — | Baseline |
| Jul 2025 | MRI, post-contrast T1 | 5.5 | −33% | nadir | Partial response |
| Dec 2025 | MRI, post-contrast T1 | 6.0 | −27% | +9% | Target: stable disease. Overall: PD by non-target lesion (cutaneous ulceration) |

*Response assessed by RECIST 1.1 with a single target lesion; the March 2025 MRI (longest diameter 8.2 cm) served as baseline. At December 2025 the target lesion did not meet criteria for progression (+9% vs the July nadir; below the ≥20% relative increase required, despite a 5 mm absolute increase); progressive disease was declared on the basis of unequivocal progression of a non-target lesion (cutaneous ulceration).*

*MRI, magnetic resonance imaging; PD, progressive disease; RECIST, Response Evaluation Criteria in Solid Tumors.*
