# Supplementary material for: Case Report: Treatment of an unresectable mucoepidermoid carcinoma with osimertinib
Source: Front Oncol. 2026 Sep 11;16:1927746. doi: 10.3389/fonc.2026.1927746 (PMC13612365; doi:10.3389/fonc.2026.1927746)
Supplement: Supplementary file 2 [file Table2.docx]

**CARE Checklist (2013, updated) of information to include when writing a case report**

*Manuscript: “Case Report: Treatment of an unresectable mucoepidermoid carcinoma with osimertinib.” This checklist follows the CARE guidelines (care-statement.org) and maps each item to its location in the submitted manuscript.*

| **Topic** | **Item** | **Checklist item description** | **Reported in manuscript (location)** |
| --- | --- | --- | --- |
| **Title** | 1 | The words “case report” appear in the title, with the area of focus. | Yes — “Case Report: Treatment of an unresectable mucoepidermoid carcinoma with osimertinib” (Title). |
| **Key words** | 2 | 2–5 key words identifying the diagnoses or interventions, including “case report.” | Yes — Keywords line: mucoepidermoid carcinoma; osimertinib; EGFR; tumor-agnostic therapy; salivary gland cancer; case report. |
| **Abstract** | 3a | Introduction: What is unique about this case? What does it add to the literature? | Yes — Abstract, Background. |
|  | 3b | Main symptoms and/or important clinical findings. | Yes — Abstract, Case presentation. |
|  | 3c | Main diagnoses, interventions, and outcomes. | Yes — Abstract, Case presentation and Conclusion. |
| **Introduction** | 4 | Brief background summary with references. | Yes — Introduction (refs 1–2). |
| **Patient information** | 5a | De-identified demographic and other patient information. | Yes — Case description (72-year-old man; de-identified). |
|  | 5b | Main concerns and symptoms of the patient. | Yes — Case description (submandibular mass, ~5-year history). |
|  | 5c | Medical, family, and psychosocial history including relevant genetic information; relevant past interventions and outcomes. | Yes — Case description (hypertension; family history; lifestyle; prior FNA and dental interventions). |
| **Clinical findings** | 6 | Relevant physical examination and other clinical findings. | Yes — Case description (ECOG 1; KPS 90%; 5 × 5 cm floor-of-mouth mass; BMI). |
| **Timeline** | 7 | Relevant data from this episode of care organized as a timeline (figure or table). | Yes — Table 1 (clinical timeline). |
| **Diagnostic assessment** | 8a | Diagnostic methods (e.g., physical exam, laboratory testing, imaging, questionnaires). | Yes — Diagnostic assessment: Pathology; Imaging and staging; Molecular profiling. |
|  | 8b | Diagnostic challenges (e.g., access, financial, cultural). | Yes — Molecular profiling (fusion testing unavailable in region; panel did not interrogate fusions). |
|  | 8c | Diagnosis (including other diagnoses considered). | Yes — Diagnostic assessment (low-grade MEC; incidental Gleason 6 prostate adenocarcinoma). |
|  | 8d | Prognosis (e.g., staging) where applicable. | Yes — Diagnostic assessment / Discussion (unresectable disease; prognostic factors discussed). |
| **Therapeutic intervention** | 9a | Types of intervention (e.g., pharmacologic, surgical, preventive, self-care). | Yes — Therapeutic intervention (osimertinib 80 mg/day; subsequent radiotherapy). |
|  | 9b | Administration of intervention (e.g., dosage, strength, duration). | Yes — Therapeutic intervention (80 mg once daily from 28 Mar 2025; 55 Gy in 20 fractions). |
|  | 9c | Changes in the intervention with explanations. | Yes — Response assessment and clinical course (osimertinib maintained at progression; radiotherapy added). |
| **Follow-up and outcomes** | 10a | Clinician- and patient-assessed outcomes. | Yes — Response assessment and clinical course; Patient perspective. |
|  | 10b | Important follow-up diagnostic and other test results. | Yes — Response assessment (serial MRI; CA19-9; repeat genomic panel). |
|  | 10c | Intervention adherence and tolerability (how was this assessed). | Yes — Response assessment (grade 1 folliculitis, mild asthenia; continued once-daily oral therapy). |
|  | 10d | Adverse and unanticipated events. | Yes — Response assessment (grade 1 folliculitis controlled with topical therapy; mild asthenia). |
| **Discussion** | 11a | Strengths and limitations in the management of this case. | Yes — Discussion: Strengths and limitations. |
|  | 11b | Relevant medical literature. | Yes — Discussion (refs 1–18). |
|  | 11c | Rationale for conclusions (including assessments of cause and effect). | Yes — Discussion (AREG–EGFR autocrine mechanism; transient response consistent with preclinical data). |
|  | 11d | Main take-away lessons of this case report. | Yes — Discussion: Take-away lessons. |
| **Patient perspective** | 12 | The patient should share their perspective on the treatment(s) they received. | Yes — Patient perspective. |
| **Informed consent** | 13 | Did the patient give informed consent? Provide if requested. | Yes — Declarations: Informed consent (written consent obtained). |

The CARE checklist is distributed under the Creative Commons Attribution license. Reference: Gagnier JJ, Kienle G, Altman DG, Moher D, Sox H, Riley D; CARE Group. The CARE guidelines: consensus-based clinical case reporting guideline development. BMJ Case Rep. 2013.
